# Analysis of factors influencing postoperative kinesiophobia in patients with perianal diseases based on structural equation modeling

**DOI:** 10.3389/fmed.2025.1726237

**Published:** 2026-01-12

**Authors:** Ailin He, Xingshun Zou, Mei Yuan, Xiang Que, Lan Su, Jiayi He, Lifang Mao

**Affiliations:** 1 School of Nursing, North Sichuan Medical College, Nanchong, Sichuan, China; 2 Nursing Department, Affiliated Hospital of North Sichuan Medical College, Nanchong, China; 3 Nanchong Mental Health Center of Sichuan Province, Nanchong, Sichuan, China

**Keywords:** influencing factors, kinesiophobia, pain, perianal disease, structural equation modeling

## Abstract

**Objective:**

This study aims to investigate the prevalence of postoperative motion kinesiophobia among patients undergoing perianal surgery and its influencing factors. Utilizing structural equation modeling, we explore the causal pathways and magnitude of these factors to inform the development of targeted clinical interventions. Ultimately, this seeks to reduce postoperative motion kinesiophobia levels in perianal surgery patients and enhance their quality of life.

**Methods:**

Employing convenience sampling, 441 patients undergoing perianal surgery were enrolled. A cross-sectional survey utilized a demographic questionnaire, the Tampa Scale of Kinesiophobia (TSK), the Postoperative Pain Coping Strategies Questionnaire, the Brief Illness Perception Questionnaire (B-IPQ), the General Self-Efficacy Scale (GSES), and the Social Support Scale: structural equation modeling analyzed postoperative anxiety factors and their causal pathways.

**Results:**

Survey findings from the 441 enrolled patients revealed: TSK scores: (41.57 ± 6.82); Postoperative pain coping strategies: (84.45 ± 9.14); GSES: (27.72 ± 3.96); B-IPQ (33.66 ± 11.35), the Social Support Scale score was (40.35 ± 7.41); and the pain rating was (4.63 ± 1.41). The structural equation model demonstrated good data fit: χ^2^/df = 1.888, GFI = 0.969, AGFI = 0.946, IFI = 0.927, TLI = 0.889, CFI = 0.925, RMSEA = 0.045. Pain exerted a positive influence on Kinesiophobia (β = 0.242, *p* < 0.001) and Illness Perception (β = 0.238, *p* < 0.001); self- efficacy negatively influenced Kinesiophobia (β = −0.236, *p* < 0.001), while social support negatively affected Kinesiophobia (β = −0.187, *p* < 0.001). The most significant effect on Kinesiophobia was exerted by pain intensity (total effect 0.401), producing a direct influence, followed by social support (total effect −0.318), self-efficacy (total effect −0.241), Illness Perception (total effect 0.238).

**Conclusion:**

Pain exerts the most significant influence on postoperative kinesiophobia in patients with perianal diseases, and this effect can be indirectly mediated through social support, self-efficacy, and Illness Perception. Clinically, healthcare professionals should prioritize pain management for patients and implement effective interventions to enhance self-efficacy, foster accurate Illness Perception, thereby reducing kinesiophobia symptoms and promoting postoperative recovery.

## 1 Introduction

Perianal diseases are common conditions of the digestive system, typically occurring in the anal canal and surrounding area. They primarily include hemorrhoids, perianal abscesses, anal fissures, anal fistulas, and hypertrophic anal papillae (1, 2). In recent years, shifts in lifestyle and dietary patterns have contributed to an increasing incidence of perianal diseases in China (3). Currently, surgical intervention remains the primary treatment approach (4). Due to the unique anatomical structure of the perianal region, patients often experience multiple postoperative discomforts, including pain, urinary retention, defecation difficulties, and incisional edema, with pain being the most prevalent symptom (5). Postoperative pain reduces mobility and carries the risk of diminished intestinal motility (6), thereby increasing the likelihood of postoperative constipation. Postoperative discomfort is a primary factor delaying functional rehabilitation and resumption of daily activities (7). Kinesiophobia, or fear of movement, denotes an excessive or irrational fear of physical activity arising from heightened sensitivity following painful injury (8). The Expert Consensus on Enhanced Recovery After Surgery for Benign Anal Diseases (2021) (9) recommends patients attempt ambulation on the day of surgery, a measure beneficial for reducing complications such as postoperative urinary retention, perianal edema, and fecal blood. However, the presence of kinesiophobia may impede early mobilization, negatively impacting the postoperative recovery process. Consequently, this cross-sectional study investigates the prevalence of postoperative kinesiophobia among patients with perianal diseases. Employing structural equation modeling, it analyses the current state of postoperative kinesiophobia in this population and examines the causal pathways among influencing factors. The research aims to provide theoretical foundations for developing targeted intervention programs to mitigate postoperative kinesiophobia levels and promote early functional rehabilitation.

## 2 Materials and methods

### 2.1 Study population

This study employed convenience sampling to select patients with perianal diseases admitted to the Integrated Chinese and Western Medicine Anorectal Department of a Grade A tertiary general hospital in Nanchong City between February and June 2025, who met the inclusion criteria. Inclusion criteria: ➀ Patients clinically diagnosed with perianal disease who had undergone surgical treatment; ➁ Age ≥ 18 years; ➂ Patients with normal cognitive function capable of independently completing questionnaires or providing clear responses; ➃ Voluntary participation with signed informed consent. Exclusion criteria: ➀ Patients with psychiatric disorders unable to cooperate; ➁ Patients with lower limb mobility impairments. A preliminary survey of 30 patients prior to formal study revealed a standard deviation (σ = 4.92) for postoperative kinesiophobia scores among perianal disease patients. Using the cross-sectional study formula *n* = [(u_α2_ × σ)/δ]^2^, with δ = 0.5, and consulting tables to determine u_α2_ = 1.96, the permissible error yielded *n* = (1.96 × 4.92/0.5)^2^ = 372 cases. Accounting for a 10% non-response rate, the sample size was set at 409 cases. The final sample size was 441 cases. This study protocol has been approved by the Ethics Committee of the Affiliated Hospital of North Sichuan Medical College (Approval No.: 2025ER71-1).

### 2.2 Research methods

#### 2.2.1 Survey instruments

##### 2.2.1.1 General treatment questionnaire

This study employed a self-designed questionnaire to gather the following patient information. General details comprised: age, gender, marital status, monthly household income, place of residence, type of carer, method of medical payment, and educational attainment. Disease-related details included: presence of underlying conditions, use of postoperative analgesia, analgesic efficacy, number of hospital admissions, postoperative fear of defecation, postoperative fear of dressing changes, and postoperative fear of mobility.

##### 2.2.1.2 Tampa scale of kinesiophobia

Kori et al. (8) developed the Tampa Scale of Kinesiophobia (TSK) in 1990. Subsequently adapted for Chinese use by Hu Wen (10), it exhibits a Cronbach’s alpha coefficient of 0.778. Currently employed to evaluate patients’ attitudes toward pain and physical activity, it finds extensive application in clinical diagnosis, monitoring, and prognosis. Its suitability for assessing kinesiophobia across diverse conditions has been validated (11). The scale employs a four-point Likert scale, scoring items from “Strongly disagree” to “Strongly agree” with values ranging from 1 to 4. It comprises 17 items, with items 4, 8, 12, and 16 reverse-scored. The total score ranges from 17 to 68 points, with a cut off score of 37 indicating kinesiophobia. Higher scores reflect greater levels of kinesiophobia. In this study, Cronbach’s α reached 0.800, demonstrating good reliability and validity.

##### 2.2.1.3 Brief illness perception questionnaire

The Brief Illness Perception Questionnaire (B-IPQ) was originally developed by Broadbent et al. (12). Subsequently, Sun Weiming’s team (13) adapted it into Chinese, yielding a Cronbach’s α coefficient of 0.831. Characterized by its conciseness and accessibility, this instrument was employed in the present study to assess patients’ illness perceptions. The first eight items are scored on a scale of 0–10, yielding a maximum total of 80 points. Items 3, 4, and 7 are reverse scored; higher scores indicate lower illness perception levels. Scores of 50–80 indicate poor illness perception, 30–49 indicate average perception, and < 30 indicate good perception. In this study, the scale demonstrated reliable internal consistency with a Cronbach’s α coefficient of 0.843.

##### 2.2.1.4 Postoperative pain coping strategies

The Postoperative Pain Coping Strategies Questionnaire developed by Juan et al. (14) indicates that patients with severe pain catastrophizing are more prone to fear, leading them to adopt avoidance coping strategies. This scale exhibits good reliability and validity, with a Cronbach’s α coefficient of 0.830. Consequently, this scale was adopted for the present study. The questionnaire comprises 22 items across five dimensions: reinterpretation (three items), courageous confrontation (5 items), catastrophizing (4 items), distraction (7 items), and neglect (6 items). It employs a 7-point Likert scale, where 0 denotes “never” and 6 denotes “always,” yielding a total score range of 0–132 points. Higher scores indicate more proactive coping strategies adopted by patients in response to pain. In this study, the scale demonstrated a Cronbach’s α coefficient of 0.879.

##### 2.2.1.5 General Self-Efficacy Scale

German psychologists Schwarzer et al. developed the General Self-Efficacy Scale (GSES) in 1981 (15). Chinese scholars Caikang et al. (16) undertook its adaptation for Chinese use, and it is now widely employed. The scale comprises 10 items with a Cronbach’s α coefficient of 0.87. It utilizes a 4-point Likert scale, scoring items from “completely incorrect” to “completely correct” with values ranging from 1 to 4 points. The minimum total score is 10 points, and the maximum is 40 points. Higher assessment scores indicate greater cognitive awareness and confidence in one’s capabilities. Scores of 10–20 indicate low levels, 21–30 denote moderate levels, and 31–40 signify high levels. In this study, Cronbach’s α was 0.804, demonstrating good reliability and validity.

##### 2.2.1.6 Social Support Scale

The Social Support Assessment Scale developed by Shuiyuan (17) incorporates an evaluation of support utilization in addition to objective and subjective support, demonstrating greater applicability within the Chinese cultural context. The scale comprises three dimensions: subjective support (items 1, 3, 4, 5), objective support (items 2, 6, 7), and utilization of support (items 8, 9, 10), with Cronbach’s α coefficients of 0.849, 0.825, and 0.833 respectively. Scores range from 11 to 72, with higher scores indicating greater levels of social support received. In this study, the Cronbach’s α coefficients for the three dimensions were 0.810, 0.833, and 0.871 respectively.

##### 2.2.1.7 Numeric Rating Scale

This study employed the Numeric Rating Scale (NRS) to assess pain intensity. This scoring method is straightforward and easily comprehensible, making it suitable for most patients (18). Participants indicate their current pain level by marking a number on a straight line labeled from 0 to 10. A score of 0 denotes no pain; 1–3 indicates mild pain; 4–6 signifies moderate pain; and 7–10 represents severe pain.

#### 2.2.2 Data collection methods

The Expert Consensus on Enhanced Recovery After Surgery for Benign Anal Diseases (9) advocates for patients to mobilize on the day of surgery. A study on postoperative pain following perianal surgery (19) indicated that pain peaks within the first 24 h post-operation. Prior to the formal survey, a pilot study was conducted on 30 patients. Pain scores were recorded at 4 h, 8 h, the first dressing change, 12 h, and 24 h after surgery. The results indicated that the patients experienced the most significant pain during the first dressing change, as shown in Table 1. Therefore, data collection for this study was completed at the time of the first dressing change within 24 h after the patients’ surgery. Prior to formal administration, participants were informed of the study’s objectives, significance, content, procedures, and precautions. For those unable to complete the questionnaire independently, researchers administered the survey verbally and recorded responses accordingly. Completed questionnaires were collected immediately and verified for completeness. A total of 450 questionnaires were distributed, yielding 441 valid responses, representing a 98% effective recovery rate.

**TABLE 1:** Comparison of scores at various time points after surgery.

Time	NRS
4 h	2.97 ± 0.85
8 h	3.80 ± 1.35
The first dressing change after surgery	5.33 ± 1.18
12 h	4.17 ± 1.02
24 h	3.40 ± 1.32

#### 2.2.3 Statistical methods

Data processing employed SPSS 27.0 and AMOS 24.0 software. Normally distributed quantitative data were presented as mean ± standard deviation; categorical data were described using frequency and percentage. Variable correlations were analyzed using Pearson correlation coefficients. Path relationships between variables were validated via structural equation modeling. All statistical analyses regarded *p* < 0.05 as indicative of statistically significant differences.

## 3 Results

### 3.1 General characteristics of study participants

This study included 441 patients aged 18–82 years, with a mean age of 45.38 ± 13.86 years; 239 males (54.2%) and 202 females (45.8%); the majority were Han ethnicity (98.4%); most resided in urban districts (224, 50.8%) or county towns (126, 28.6%), with the remainder living in towns (42, 9.5%) or rural areas (49, 11.1%) (see Table 2).

**TABLE 2:** Comparison of postoperative dyskinesia scores among perianal disease patients by demographic characteristics and disease features (*n* = 441).

Variables		Total, n(%)	Kinesiophobia	*t*/*F*	*P*
Age	<40	170(38.5)	42.46 ± 6.99	2.387	0.093
	40–40	210(47.6)	41.0 ± 6.96		
	> 60	61(13.8)	41.02 ± 5.60		
Gender	Male	239(54.2)	41.72 ± 6.70	0.273	0.602
	Female	202(45.8)	41.38 ± 7.19		
Ethnic group	Han Chinese	434(98.4)	41.50 ± 6.80	2.612	0.107
	Ethnic minorities	7(1.6)	45.71 ± 6.86		
Place of residence	City center	224(50.8)	41.50 ± 6.52	0.061	0.98
	County town	126(28.6)	41.51 ± 7.20		
	Town	42(9.5)	41.67 ± 7.38		
	Rural	49(11.1)	41.93 ± 7.17		
Educational attainment	Primary school and below	66(15.0)	40.67 ± 6.29	0.698	0.554
	Junior School	111(25.2)	41.50 ± 7.44		
	Secondary school	112(25.4)	41.45 ± 7.15		
	University and above	152(34.5)	42.10 ± 6.86		
Marital status	Unmarried	50(11.3)	44.58 ± 7.50	5.856	0.003
	Married	387(87.8)	41.16 ± 6.66		
	Divorced	4(0.9)	43.75 ± 9.11		
Occupation	Peasant	58(13.2)	42.24 ± 6.77	3.606	0.003
	Full-time employment	150(34.0)	41.12 ± 6.39		
	Retirement	42(9.5)	38.57 ± 6.75		
	Unemployed	133(30.2)	42.24 ± 6.89		
	student	21(4.8)	45.48 ± 7.80		
	Others	37(8.4)	41.08 ± 7.12		
Medical payment methods	Employee basic medical insurance	173(39.2)	40.71 ± 6.50	2.287	0.103
	Urban resident medical insurance	263(59.6)	42.11 ± 7.02		
	Others	5(1.1)	42.80 ± 8.70		
Comorbidities	Yes	92(20.9)	40.96 ± 7.40	0.921	0.338
	No	349(79.1)	41.73 ± 6.70		
Analgesic use	Yes	145(32.9)	39.50 ± 6.29	20.576	<0.01
	No	296(67.1)	42.58 ± 6.90		
Whether to retain the urinary catheter	Yes	38(8.6)	44.47 ± 6.30	7.587	0.006
	No	403(91.4)	41.29 ± 6.85		
Number of hospital admissions	1	368(83.4)	41.64 ± 6.80	0.279	0.598
	≥ 2	73(16.6)	41.18 ± 6.86		
Fear of postoperative defecation	Yes	242(54.88)	45.06 ± 5.85	202.816	<0.01
	No	199(45.12)	37.32 ± 5.46		
Fear of postoperative dressing changes	Yes	334(75.74)	43.65 ± 6.08	178.933	<0.01
	No	107(24.26)	35.06 ± 4.76		
Fear of postoperative activity	Yes	279(63.27)	44.44 ± 5.87	190.443	<0.01
	No	162(36.73)	36.62 ± 5.48		
First dressing change NRS	Mild pain	92(20.86)	37.55 ± 6.25	32.041	<0.01
	Moderate pain	311(70.52)	42.11 ± 6.42		
	Severe pain	38(8.62)	46.87 ± 6.78		

### 3.2 the level of kinesiophobia, illness perception, pain coping strategies, self-efficacy, and social support status in patients with perianal diseases after surgery

Postoperative kinesiophobia scores for patients with perianal diseases were (41.57 ± 6.82) points; the total postoperative pain coping strategy score was (84.45 ± 9.14) points, with specific scores across dimensions as follows: Ignoring (19.33 ± 6.03); Catastrophizing (9.18 ± 4.46); Distraction (26.93 ± 4.85). Patients’ self-efficacy and Illness Perception scores were (27.72 ± 3.96) and (33.66 ± 11.35) respectively. The total social support score was (40.35 ± 7.41), comprising subjective support (10.18 ± 2.37), objective support (22.30 ± 5.56), and support utilization (7.87 ± 2.10). Finally, the numerical pain rating was (4.63 ± 1.41).

### 3.3 Univariate analysis results for postoperative kinesiophobia in patients with perianal diseases

As shown in Table 1, statistically significant differences (*p* < 0.05) were observed in mobility anxiety scores among patients with varying marital statuses, occupations, analgesic usage, indwelling urinary catheter status, and fears regarding postoperative bowel movements, dressing changes, or ambulation, as well as those with differing pain scores during the first postoperative dressing change.

### 3.4 Correlation Analysis results for postoperative kinesiophobia, pain coping strategies, self-efficacy, social support, illness perception, and pain scores in patients with perianal diseases

Correlation analysis revealed that following surgical intervention for perianal disorders, patients exhibited negative correlations between anxiety levels and self-efficacy and social support; but positively correlated with brief illness perception and pain score. Furthermore, there was no significant correlation between the level of kinesiophobia and pain coping strategies (see Table 2 for details).

### 3.5 Construction of the structural equation model

#### 3.5.1 Model construction

**FIGURE 1:**
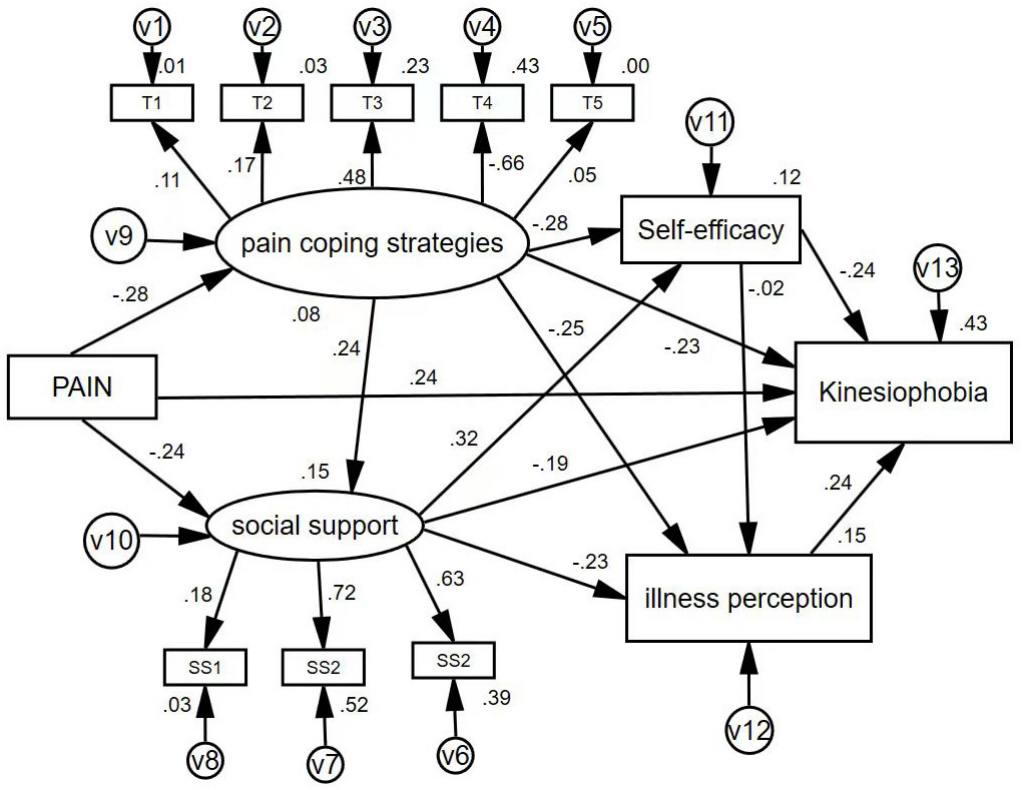
The definitive construction equation model with standardized paths. SS1, subjective support; SS2, objective support; SS3, utilization of social support; T1, re-explained; T2, face it bravely; T3, neglect; T4, catastrophization; T5, distraction.

Integrating the motion fear model with research hypotheses, AMOS software was employed to construct an initial model. This model treated postoperative motion fear in patients with perianal diseases as the dependent variable, pain scores as the independent variable, and self-efficacy, pain coping strategies, illness Perception, and social support as mediating variables. Maximum likelihood estimation was used for model fitting, as illustrated in Figure 1. Model fit statistics were: χ^2^/df = 1.888, GFI = 0.969, AGFI = 0.946, IFI = 0.927, TLI = 0.889, CFI = 0.925, RMSEA = 0.045. All model fit indices fall within the acceptable range, indicating satisfactory model fit. Within this model, patients’ self-efficacy, illness Perception, and social support collectively explain 42.6% of the variance in kinesiophobia.

#### 3.5.2 Path results

The factors influencing kinesiophobia, ranked from greatest to least impact, are pain intensity, social support, self-efficacy, and illness Perception, with specific data shown in Table 3. Path analysis from the structural equation model shows that pain intensity has a significant direct positive effect on akinesia (β = 0.242, *p* < 0.001), making it the most influential direct factor. At the same time, social support has a negative effect (β = −0.187, *p* < 0.05), indicating higher social support is associated with lower levels of kinesiophobia. Social support also indirectly affects kinesiophobia through pathways involving self-efficacy and illness Perception. Self-efficacy has a direct negative impact on kinesiophobia (β = −0.236, *p* < 0.001) and influences it indirectly via illness cognition. Additionally, illness cognition itself significantly and positively predicts kinesiophobi (β = 0.283, *p* < 0.001). However, the path coefficient for pain coping strategies was *p* > 0.05, which means it is not statistically significant (for more details, see Figure 1 and Tables 4, 5).

**TABLE 3:** Correlation between various variables in patients with perianal diseases following surgery (*n* = 441).

Variable	Kinesiophobia	Self-efficacy	Pain coping strategies	Illness Perception	Social support	NRS
Kinesiophobia	1				
Self-efficacy	−0.262**	1			
Pain coping strategies	−0.093	−0.035	1		
Illness perception	0.399**	−0.027	−0.055	1	
Social support	−0.399**	0.164**	0.201**	−0.236**	1
NRS	0.404**	−0.079	−0.035	0.101*	−0.243**	1

*p < 0.05,

**p < 0.01.

**TABLE 4:** Path analysis of structural equation modeling on influencing factors of kinesiophobia in patients with perianal diseases after surgery.

Hypothesized relationship	β	SE	CR	*P-*value
A→F	0.242	0.207	5.643	***
B→F	−0.974	0.325	−2.999	0.003
C→F	0.143	0.027	5.399	***
D→F	−0.408	0.076	−5.346	***
E→F	−4.466	2.865	−1.559	0.119

***p < 0.001. A, Pain; B, Social Support; C, illness Perception; D, self-efficacy; E, Pain Coping Strategies; F, kinesiophobia.

**TABLE 5:** The direct, indirect, and total effects of variables in the definitive model.

Pathways	Standardized direct effects	Standardized indirect effects	Standardized total effects
A→F	0.242	0.159	0.401
B→F	−0.187	−0.131	−0.318
C→F	0.283	–	0.238
D→F	−0.236	−0.005	−0.241
E→F	−0.227	−0.068	−0.295

A, Pain; B, Social Support; C, illness Perception; D, self-efficacy; E, Pain Coping Strategies; F, kinesiophobia.

## 4 Discussion

### 4.1 The influence of self-efficacy on kinesiophobia

Based on the results of the structural equation modeling analysis, self-efficacy can both directly influence kinesiophobia levels and exert indirect effects through other pathways, with a total effect of 0.241. Self-efficacy represents an individual’s perception and belief regarding their capacity to adopt appropriate behaviors when confronting environmental challenges, reflecting their ability to manage various pressures (16). The direct positive relationship observed between pain catastrophizing and kinesiophobia indicates that self-efficacy may also exert a direct influence on kinesiophobia within the context of pain management. Relevant research demonstrates (20) that higher levels of self-efficacy among patients correlate with reduced negative impacts from pain. Furthermore, high self-efficacy may serve as a protective factor against adverse effects arising from disease progression. Feng Cencen’s research (21) indicates that patients with low self-efficacy struggle to cope with challenges and setbacks, lack confidence in confronting difficulties, and deny their own agency. Consequently, they fail to proactively engage in early postoperative activities. Patients with perianal diseases experience intense postoperative pain, particularly exacerbated during bowel movements, leading to significant activity limitations and severe physical and emotional distress (22). Path analysis results from this study indicate that self-efficacy mediates the relationship between pain and kinesiophobia. Consequently, assisting patients in effectively managing postoperative pain and reducing cognitive distortions regarding pain not only directly diminishes pain perception but also enhances self-efficacy. Through this self-efficacy pathway, establishing robust social support systems and improving disease cognition can help mitigate the occurrence of kinesiophobia.

### 4.2 The influence of social support on kinesiophobia

Among the various pathways influencing the development of kinesiophobia in this study, social support exhibited the smallest effect size. Social support exerted a significant direct negative influence on kinesiophobia, though the magnitude of this direct effect was relatively limited. As a multidimensional form of support from family and social networks, social support fundamentally enhances an individual’s capacity to cope with challenges (17). This study found a negative correlation between social support and fear of movement. This aligns with theoretical expectations: higher social support enhances patients’ confidence and adherence to post-operative activities, thereby directly alleviating fear of movement. This result parallels findings by Marmouta et al. (23), indicating that social support not only improves patients’ performance in daily activities but also enhances emotional perceptions of their own health during rehabilitation. Among the study participants, social support primarily originated from family members. Patients exhibited significant dependence on relatives’ post-surgery, likely due to the effective alleviation of negative emotions through familial reassurance and timely provision of both psychological and material support. Self-efficacy serves as a crucial cognitive mechanism regulating patients’ behavioral activities. The sense of belonging and security derived from social support, combined with enhanced self-efficacy, collectively form an important mediating pathway for reducing agoraphobia. Therefore, promptly recognizing the social support needs of Kinesiophobia patients and establishing effective social support systems can help patients adjust their attitudes toward coping with the condition, enabling them to confront the onset of Kinesiophobia with a positive mindset.

### 4.3 The influence of illness perception on kinesiophobia

The findings of this study indicate that Illness Perception exerts a direct positive influence solely upon kinesiophobia. Higher scores on the Illness Perception scale signify poorer understanding of disease knowledge among patients, correlating with correspondingly elevated kinesiophobia scores. According to Leeuw et al. (24) modified fear-avoidance model, when individuals possess unclear Illness Perception, they are prone to develop anxiety and avoidance emotions, ultimately forming avoidance behaviors. Supporting evidence is provided by relevant research (25): a survey on early postoperative recovery among patients with perianal diseases revealed that those with higher Illness Perception demonstrated significantly superior recovery quality compared to those with poorer cognition. Thus, greater mastery of disease knowledge correlates with higher compliance in early mobilization and more pronounced self-care outcomes. The Health Belief Model (26) corroborates this perspective from another angle, positing that “beliefs can alter behavior.” Accurate Illness Perception facilitates shifts in patients’ attitudes and beliefs toward health, thereby promoting active engagement in rehabilitation activities. However, clinical observations reveal that the vast majority of patients perceive healthcare professionals as primarily responsible for their health, overlooking their own crucial role in health promotion (26). This underscores the need for nursing staff to enhance health education during hospitalization, improve patients’ illness Perception, and reinforce health promotion. Such efforts should help patients cultivate a positive health consciousness and proactively seek ways to foster and sustain health-enhancing behaviors.

### 4.4 Impact of pain severity on kinesiophobia

Path analysis revealed that among multiple factors influencing postoperative Kinesiophobia in perianal disease patients, pain severity exhibited the most pronounced total effect value, with an effect coefficient of 0.401. Path relationships indicate that pain not only directly influences Kinesiophobia but also affects it through three mediating pathways: social support, self-efficacy, and illness Perception. This study confirms that pain primarily exerts a direct effect on kinesiophobia. Consequently, to effectively reduce kinesiophobia levels in patients with perianal diseases, healthcare professionals must not only closely monitor pain status and promptly implement effective pain relief measures, but also enhance disease cognition through health education, cultivate self-efficacy, and mobilize family members to provide emotional support. This comprehensive approach alleviates patients’ kinesiophobia. Self-efficacy proves particularly crucial, directly determining whether patients will proactively take steps to overcome pain-related fears (24). Consequently, nursing staff should dynamically assess postoperative pain levels, develop personalized pain management strategies addressing both physiological and psychological factors, and bolster patients’ confidence in overcoming pain. This involves guiding patients toward a positive outlook on pain, actively providing pain-relief strategies, and encouraging early engagement in rehabilitation activities.

### 4.5 Impact of pain coping strategies on kinesophobia

In this study, postoperative pain coping scores for patients with perianal diseases averaged 9.52 ± 4.61 points. However, path analysis revealed no statistically significant effect of pain coping on the occurrence of postoperative kinesophobia in these patients. This finding diverges from the expected hypothesis and may be due to several factors. First, Leeuw et al. (24) proposed in the fear-avoidance model that chronic pain induces associated fears, leading to negative coping mechanisms. Psychological research on pain-related phenomena has also shown that anxiety and fear play a more significant role in chronic pain. However, postoperative pain in perianal disease is characterized by defecation-related paroxysmal pain (27). Wei (19) research similarly indicates that postoperative pain in patients with perianal diseases peaks within 24 h. The negative emotions caused by pain lead to reduced self-efficacy (28). Among patients with low self-efficacy, the impact of pain intensity on fear of movement becomes more pronounced, and the mediating role of pain coping is overlooked, thereby weakening this pathway. In this study, 2 h after postoperative patients returned to the ward, their responsible nurses provided health education to the patients and their family members, explaining pain relief methods to the patients. Meanwhile, all postoperative patients in the study department received traditional Chinese medicine interventions such as auricular point pressing to alleviate pain. Additionally, the patients were guided to adopt a progressive approach to postoperative activities.

Therefore, these interventions might have narrowed the individual differences in patients’ pain coping strategies and early activity behaviors, which resulted in the weakened impact of kinesiophobia in the model.

## 5 Summary

The occurrence of postoperative kinesophobia in patients with perianal disorders is influenced by multiple factors, ranked in order of significance as follows: pain, social support, self-efficacy, and illness Perception. Consequently, in clinical nursing practice, healthcare professionals should identify the factors influencing the development of kinesophobia and their pathways of action to implement timely and effective interventions, thereby exerting a positive influence through these pathways. Concurrently, clinicians must monitor patients’ overall rehabilitation progress, devising personalized and progressive post-operative activity programs. Nursing staff should provide ongoing health education and support, while rehabilitation therapists deliver specialized exercise supervision and safety guidance. Through collaborative efforts, these professionals assist patients in breaking the vicious cycle of fear-avoidance associated with kinesiophobia. This study focused on the impact of acute postoperative pain on patients. However, the investigation revealed that postoperative pain has a persistent effect. Consequently, subsequent research should further explore the influence of prolonged postoperative pain on patients’ daily activities and quality of life. Given that this study adopted a cross-sectional design, which cannot determine the temporal sequence and causal relationship between variables, we will further explore the interaction between kinesiophobia and related factors at multiple time points before and after surgery in subsequent research, so as to provide data support for evaluating the long-term effects of intervention measures. This study adopted a convenience sampling method and was conducted at a single medical center, so the sample size has certain limitations and cannot fully represent other patient groups. Therefore, in subsequent research, we can carry out a multicenter and randomized sampling design, recruit patients from medical institutions of different levels in various regions, and improve the generalizability of the research results.

## Data Availability

The raw data supporting the conclusions of this article will be made available by the authors, without undue reservation.
